# Mindfulness-Based Self-Management Program Using a Mobile App for Patients With Pulmonary Hypertension: Single-Arm Feasibility Study

**DOI:** 10.2196/79639

**Published:** 2026-02-04

**Authors:** Yuka Takita, Junko Morishita, Sunre Park, Ayumi Goda, Takumi Inami, Hanako Kikuchi, Takashi Kohno, Masaharu Kataoka, Daisuke Fujisawa

**Affiliations:** 1 Department of Nursing, Faculty of Medical Technology, Teikyo University, 2-11-1, Kaga, Itabashi-ku, Tokyo, 173-8605, Japan, 81 3 3964 1211 ext 46114, 81 3 3964 3365; 2 Department of Nursing, National College of Nursing, Japan Institute for Health Security, Tokyo, Japan; 3 Department of Nursing, Faculty of Nursing and Medical Care, Keio University, Tokyo, Japan; 4 Department of Cardiovascular Medicine, Faculty of Medicine, Kyorin University, Tokyo, Japan; 5 The Second Department of Internal Medicine, University of Occupational and Environmental Health, Kitakyushu, Japan; 6 Division of Quality Assurance Programs, National Cancer Center Institute for Cancer Control, Tokyo, Japan

**Keywords:** mindfulness, mindfulness-based intervention, pulmonary hypertension, self-management, digital health

## Abstract

**Background:**

Mindfulness-based interventions have been applied across various chronic illnesses, but no tailored program exists for individuals with pulmonary hypertension (PH).

**Objective:**

This study aimed to develop and evaluate the feasibility of a mindfulness-based self-management program for patients with PH, delivered online to accommodate their limited mobility.

**Methods:**

A single-arm pre-post study was conducted using an 8-session, weekly videoconference program incorporating PH self-management education and elements of mindfulness-based cognitive therapy. A mobile app linked to an Apple Watch was used to support symptom monitoring and mindfulness awareness. Outcomes included PH-related symptoms, quality of life (emPHasis-10), depression (Patient Health Questionnaire-9 [PHQ-9]), anxiety (Generalized Anxiety Disorder 7-item scale [GAD-7]), resilience (Connor-Davidson Resilience Scale [CD-RISC]), and loneliness (UCLA Loneliness Scale–short version). Assessments occurred at baseline, week 4, and program completion. Exit interviews explored perceived changes and experiences.

**Results:**

Twelve participants (mean age 41.8, SD 10.5 years; range 26‐56 years) were enrolled, and 9 completed the program (75% retention). Participants valued the online format and Apple Watch integration, while noting a need for optional on-demand sessions. Qualitative analysis identified themes such as increased self-awareness, use of meditation for pain management, and enhanced self-compassion. Quantitative analysis showed significant changes across 3 time points (baseline, week 4, and week 8) for emPHasis-10 (*χ*²_₂_=9.74; *P*=.008) and CD-RISC (*χ*²_₂_=7.27; *P*=.03). Trends toward change were observed for PHQ-9 (*χ*²_₂_=4.75; *P*=.09) and GAD-7 (*χ*²_₂_=5.07; *P*=.08), but week 12 data were limited (n=5). No significant changes in loneliness were observed.

**Conclusions:**

The program appeared to support patients with PH in managing symptoms and emotions and suggested potential improvements in quality of life. These preliminary findings warrant evaluation in a future randomized controlled trial.

## Introduction

Pulmonary hypertension (PH) is a progressive disease characterized by shortness of breath as the primary symptom. Although recent advances in treatment have dramatically improved the prognosis of the disease [1], patients with PH may face various physical symptoms and may experience limitations in their activities and social roles. Self-management at home has become increasingly complex owing to the use of medications with various mechanisms of action and with various routes of administration [2]. Furthermore, side effects of pulmonary vasodilative medications, such as headache, jaw pain, plantar pain, diarrhea, and nausea, may occur. These symptoms are more pronounced in continuous intravenous and subcutaneous infusion therapies. There is a certain level of risk for sudden death in severe PH cases with right heart failure; thus, patients with PH are forced to live with uncertainty.

Therefore, it is not surprising that patients with PH tend to experience high anxiety and depression. Past studies demonstrated that 21.9%‐56% of PH patients are comorbid with depressive symptoms [3-14], 10.7%‐62% with anxiety symptoms [3-6,8,12,13], and 27.6%‐40% with stress-related symptoms [4,11]. These findings suggest the need for psychological care for patients with PH.

Mindfulness has been described as “paying attention in a particular way—on purpose, in the present moment, and nonjudgmentally” [15]. Mindfulness-based interventions have gained increasing attention as a method of psychological care for patients with physical illnesses. The two major programs are mindfulness-based stress reduction (MBSR) [16] and mindfulness-based cognitive therapy (MBCT) [17]; however, flexible modifications of the program to fit with target populations, such as providing information on nutrition [18], grief care [19], and advance care planning [20,21], have been implemented.

Conventional mindfulness-based programs have been delivered on a face-to-face basis; however, in recent years, web-based programs have been developed and implemented quite widely [22,23]. In the field of cardiovascular disease, mindfulness interventions are being developed for patients with coronary artery disease and heart failure [24-26].

However, to the best of the authors’ knowledge, there has been no mindfulness-based program that has been developed specifically for PH. Modification of the program may be needed for patients with PH to avoid physical overload that may worsen right ventricular function. For example, yoga, a standard component of MBSR and MBCT, could lead to cardiopulmonary overload. Furthermore, since patients with PH often have limited mobility, web-based programs, instead of on-site programs, are likely to be preferred.

Therefore, in this study, we developed and tested the feasibility of an online mindfulness-based self-management program for patients with PH using a smartphone app on Apple Watch. The intervention aimed to improve the quality of life (QOL) and resilience and to reduce depression, anxiety, and pain (a side effect of the treatment) of patients with PH.

## Methods

### Study Design

This study used a mixed methods, single-group, pre-post design.

### Participants

#### Inclusion and Exclusion Criteria

The inclusion and exclusion criteria are shown in Textbox 1.

Textbox 1. **Inclusion and exclusion criteria.**

The inclusion criteria for this study were as follows:

- A confirmed diagnosis of pulmonary hypertension (PH) [27].
- Age 20‐75 years.
- The ability to attend at least six of the eight 60-minute online sessions.
- The ability to operate the self-administered mobile app on an iPhone or iPad and an Apple Watch.

The exclusion criteria were as follows:

- Severe physical symptoms, including but not limited to decompensated right-sided heart failure, as assessed by the attending physician.
- Patients with an active psychiatric disorder who were currently under psychiatric care or receiving psychotropic treatment. These individuals were excluded for safety reasons, as the program was delivered entirely online, and timely in-person support could not be ensured if psychological distress or other adverse emotional reactions were to occur during the intervention.
- Cognitive impairment or other conditions that, in the attending physician’s judgment, would make it difficult for the patient to understand or participate in the program, based on routine clinical assessments and information in medical records.
- Individuals deemed by their attending physicians to be clinically unstable or otherwise inappropriate for participation.
- Individuals who had previously participated in structured mindfulness-based programs such as mindfulness-based cognitive therapy (MBCT) or mindfulness-based stress reduction (MBSR).

Individuals aged 18‐19 years were not included, because at the time this study was initiated (December 2020), persons ages 20 years and younger were not considered adults for the purpose of providing independent informed consent under Japanese ethical guidelines, and thus, were unable to consent without guardian approval [28].

#### Recruitment and Screening Procedures

**Figure 1.**
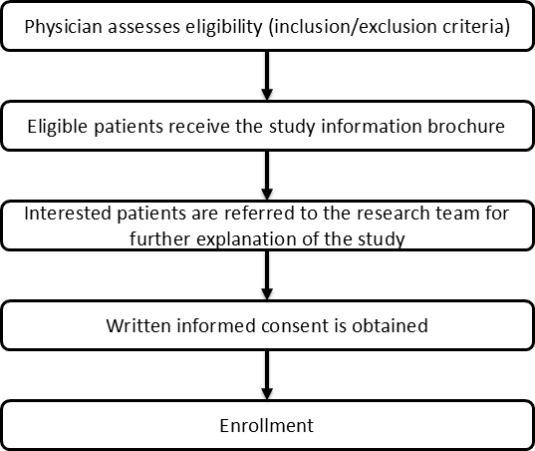
Flowchart illustrating the recruitment and screening.

Participants were recruited through a structured, stepwise process. First, physicians screened patients during routine clinical visits to ensure they met the inclusion criteria and did not meet any exclusion criteria. Eligible patients were then provided with an informational brochure describing the study. Patients who expressed interest were referred to the research team, who provided a detailed explanation of the study procedures. Written informed consent was obtained from all patients before enrollment. The overall recruitment and screening process is shown in Figure 1.

#### Sample Size Justification

As this was a feasibility study, the primary aim was to evaluate acceptability, adherence, and operational feasibility rather than to test statistical efficacy; therefore, a formal power calculation was not required. We chose a target sample size of approximately 12 participants, which is comparable to previous feasibility studies of meditation or mindfulness-based interventions in cardiovascular populations that enrolled about 10‐15 participants [29,30].

### Intervention

#### Program Contents

**Figure 2.**
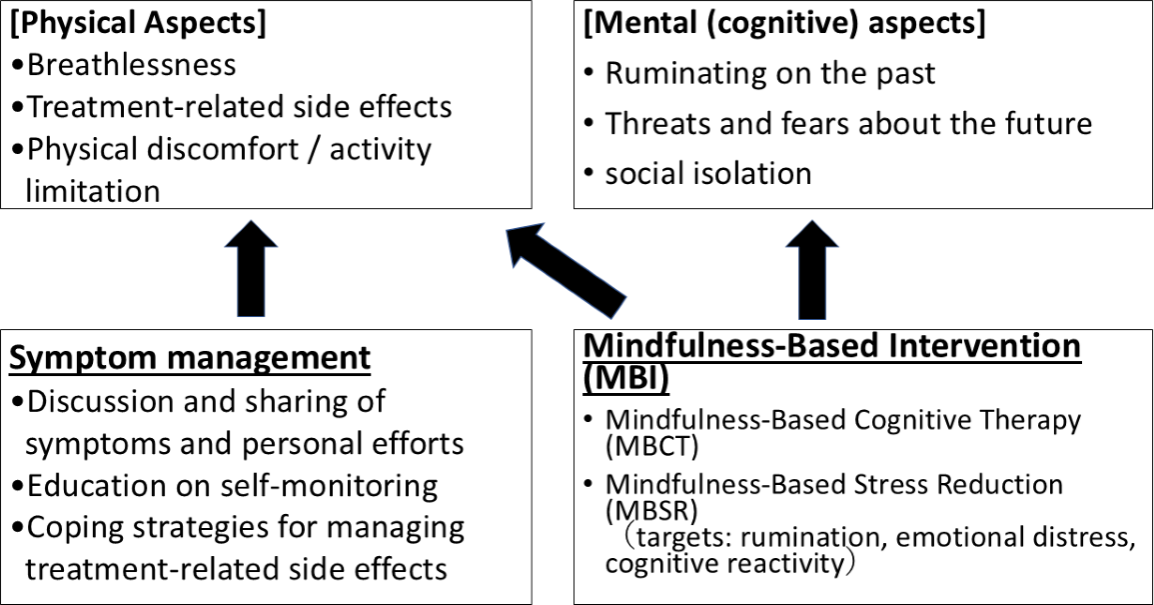
Conceptual framework of the mindfulness-based intervention program for patients with pulmonary hypertension.

The conceptual framework and content of the program are presented in Figure 2 and Table 1. We developed this program based on the findings of our previous research, which showed that the key elements of distress for patients with PH were the loss of the past and the threat of disease progression, including rumination about the past and concern about the future [14]. The fundamentals of the intervention were based on MBCT, which addresses rumination and has been proven effective for both physical and psychological symptoms in patients with critical illnesses [17]. Furthermore, we added psychoeducation and self-management skill-building as essential components, as the distress of patients with PH derives from PH-specific symptoms (eg, breathlessness) and the adverse effects (eg, pain or nausea) and difficulties associated with the treatment (eg, home oxygen therapy).

**Table 1.** Program content structured based on the results of previous research.

Elements of distress	Program content and expected effects
“Isolation from my surroundings”	Prevent isolation and reduce loneliness by regularly connecting with others and sharing thoughts and feelings through weekly online meetings.
“Loss of myself” and “Fear of illness progression or deterioration”	By reflecting on their physical and mental conditions through mindfulness practice and homework, participants become aware of their thought patterns and feelings, such as loss of abilities, regret regarding the past, and anxiety about the future; promoting meta-cognition stops rumination, thereby alleviating mood swings and anxiety.
“Hassle associated with oxygen therapy,” “Suffering from side effects,” and “Rumination on illness due to breathlessness”	Learning and sharing the basic knowledge and practical tips required for self-management and enhancing the ability to self-manage activities, shortness of breath, side effects, and other issues.

#### Program Structure

Table 2 presents the outline of the program. The basic structure of the program was similar to that of MBCT. To lessen the potential physical and psychological burden of the patients, we adapted an online format. We shortened the length of each session to 1 hour. We eliminated yoga, which has been supposed to be an integral component in conventional MBCT, for safety reasons, since patients with PH are at risk of circulatory collapse owing to direct right ventricular stress caused by increased pulmonary artery pressure, which could be exacerbated by physical exercise.

**Table 2.** Schedule and homework for the self-management mindfulness program for patients with pulmonary hypertension.

Session	Theme	Contents	Homework
1	Start the program	What is mindfulness? How to use the self-management app? Short breathing meditation	Using self-management app
2	Become aware of automatic reactions	Short breathing meditation Symptom management of pulmonary hypertension	Mindful daily living
3	Focus on your body	Body scan (meditation) Cognitive strategies for managing treatment-related side effects	Body scan
4	Focus on breathing	Mindfulness meditation of breathing and body Pleasant and unpleasant mode	Pleasant and unpleasant life diary
5	Focus on your body and adjust it	Short meditation Adjustment of pulmonary hypertension activities	Meditation Focus on your pulse during activity
6	Thoughts are not facts	Mindfulness meditation with sound and thought Cognitive strategies for managing thoughts and emotions (guided imagery practice) 3-step breathing space method	Using 3-step breathing space method when feeling uncomfortable
7	Caring for yourself	Compassion meditation Positive habits	Meditation Appreciation list
8	Use skills for your future	Meditation Reflection on the past Looking toward the future	—^a^

a Not applicable.

The program was delivered in a group format once a week over a period of 8 weeks via video conference. The program was facilitated by the lead author, a nurse with 6 years of experience in practice and the teaching of mindfulness interventions. Each session lasted 60 minutes, which included 10- to 30-minute meditation practice, which was facilitated with the facilitator. At the beginning of each session, a reflection on homework was conducted, and an inquiry session was held after each meditation practice. Homework included daily mindfulness practice (eg, breathing meditation and body-scan meditation) and reflection journaling on emotional and physical experiences. Video lectures on the meaning of mindfulness, the pleasant and unpleasant mode, and mindfulness meditation were provided by a clinical psychologist and a nurse who had been a mindfulness provider for over 10 years. Pleasant mode and unpleasant mode involve mindfully reflecting on pleasant and unpleasant experiences in daily life, noting the physical sensations, emotions, and thoughts that arose at the time, thereby cultivating awareness.

**Figure 3.**
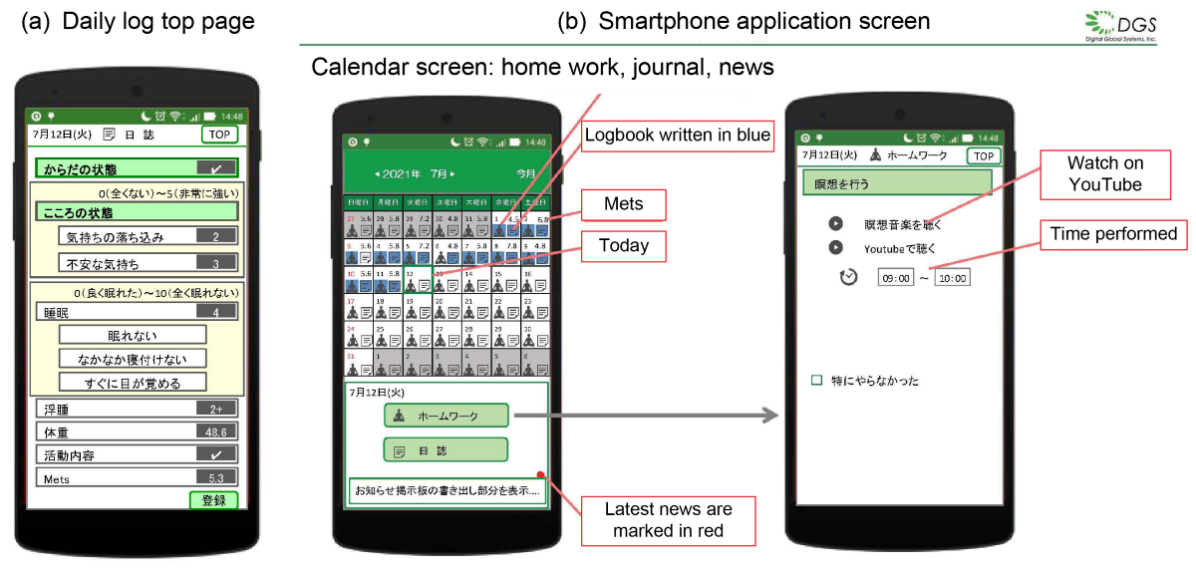
Screenshots of the self-management smartphone app used in the study.

To facilitate self-management skills, we developed a mobile app, which enabled users to record their daily physical conditions and to monitor their activity status using an Apple Watch (Figure 3). The mobile app (Self-management App, developed in collaboration with DGS Co Ltd) stored data on secure encrypted servers; all identifying information was anonymized and replaced by unique participant codes. For participants who did not own an Apple Watch, a device was lent to them free of charge for use during the study period. Participants were advised to use this mobile app throughout the program.

### Outcome Measures

#### Demographic Data

Demographic and clinical information, including age, sex, diagnosis, disease classification (World Health Organization functional class), duration of illness, and treatment status, were collected at baseline through participants’ self-report and review of medical records. Demographic information was obtained to characterize the study sample and was used only for descriptive analyses.

#### Feasibility and Acceptability

The feasibility of the intervention was evaluated with participation and completion rates for the program. Acceptability was assessed by collecting and evaluating feedback from the program through interviews. The interviews included questions about changes in symptoms, self-management skills, and mental health conditions before and after the program. Furthermore, the participants were asked to describe the perceived benefits of the program, what should be changed, what they liked and disliked, and obstacles and facilitators for implementation of the program.

#### Secondary Outcomes

Secondary outcome measures comprised emPHasis-10, a disease-specific patient-reported outcome measure for evaluating the QOL of patients with PH [31]; the Patient Health Questionnaire (PHQ-9) for depression [32]; the Generalized Anxiety Disorder 7-item (GAD-7) scale [33]; the Connor-Davidson Resilience Scale (CD-RISC), a measure of one’s ability to recover from various difficulties such as illness, emotional pressure, and pain [34]; and the UCLA Loneliness Scale-short version [35]. In addition, interviews were conducted regarding changes in awareness and behaviors related to self-management. These measures were obtained at baseline, 4 weeks after the program, and at the end of the program. The questionnaire was administered at four time points (baseline, week 4, week 8, and week 12). All questionnaires were provided in paper format, completed by participants at each time point, and returned by mail.

### Analytical Methods

Content analysis was used to analyze the qualitative data from the interviews [36]. The characteristics and results of the participants were summarized using descriptive statistics, with median (range) for continuous variables and frequency (percentage) for categorical variables. The score changes between time points for each participant were plotted as line graphs for each scale. The Friedman test was conducted to evaluate the differences in scores across time points. This nonparametric test was chosen owing to its suitability for comparing repeated measures or related samples without assuming normality. Significance was set at *P*<.05. IBM SPSS (version 28) for Windows was used for all statistical analyses. MAXQDA (VERBI Software GmbH) was used for qualitative data analysis.

### Ethical Considerations

This study was approved by the ethics review committees of Tokyo Kasei University and Kyorin University School of Medicine (SKE2020-11, number 1631) and was conducted in accordance with the ethical standards of the Declaration of Helsinki and relevant national guidelines.

All participants received a written explanation of the study’s purpose, procedures, potential risks, and their right to withdraw at any time without disadvantage. Written informed consent was obtained from all participants prior to study participation.

To ensure privacy and confidentiality, all data were anonymized and assigned participant identification codes at the time of collection. No personally identifiable information was included in the datasets used for analysis. Data were securely stored on a password-protected hard disk drive.

Participants did not receive monetary compensation but were provided with the mindfulness program free of charge as part of the study.

No identifiable images or personal information of participants are included in this paper or the supplementary files.

The study was registered in the UMIN Clinical Trials Registry (UMIN000044075).

## Results

### Participant Characteristics

Participants’ baseline characteristics are shown in Table 3.

**Table 3.** Participant characteristics at baseline (n=12).

Characteristics	Results
Age (years), mean (SD)	41.8 (10.5)
Age (years), range	26‐56
Sex, n (%)	
Male	3 (25)
Female	9 (75)
Program completed, n (%)	9 (75)
PH^a^ type, n (%)	
PAH^b^	11 (92)
CTEPH^c^	1 (8)
MeanPAP^d^ (mmHg), median (range)	32.5 (20-47)
Treatment (pharmacological therapy), n (%)	
Epoprostenol (IV^e^)	5 (42)
Treprostinil (IV)	2 (2)
Treprostinil (SC^f^)	1 (8)
Only oral medicine^g^	4 (33)
Interventional history, n (%)	
Post BPA^h^	1 (8)
Therapeutic support, n (%)	
Oxygen therapy	3 (25)
Symptoms, n (%)	
Dyspnea on exertion	10 (83)
Fatigue	10 (83)
Palpitations	3 (25)
Pain	8 (67)
Nausea	3 (25)
Diarrhea	5 (42)
QOL^i^ (emPHasis-10^j^), mean (SD), range	33.6 (10.1), 16‐46
Depression (PHQ-9^k^), mean (SD), range	14.1 (6.6), 2‐24
Anxiety (GAD-7^l^), mean (SD), range	11.1 (5.5), 1‐18
Resilience (CD-RISC^m^), mean (SD), range	17.8 (8.0), 3‐29
Loneliness (UCLA Loneliness Scale), mean (SD), range	26.6 (5.9), 13‐34

a PH: pulmonary hypertension.

b PAH: pulmonary arterial hypertension.

c CTEPH: chronic thromboembolic pulmonary hypertension.

d MeanPAP: mean pulmonary arterial pressure.

e iv: intravenous injection therapy.

f sc: subcutaneous injection therapy.

g Including the 1 participant with post-BPA status.

h BPA: balloon pulmonary angioplasty (only applies to patients with CTEPH).

i QOL: quality of life.

j emPHasis-10: disease-specific patient-reported outcome (PRO) measures for evaluating quality of life in patients with pulmonary hypertension.

k PHQ-9: Patient Health Questionnaire-9.

l GAD-7: Generalized Anxiety Disorder 7-item scale.

m CD-RISC: Connor-Davidson Resilience Scale.

### Feasibility

Of the 12 participants who agreed to participate in this study, 9 completed the program (75% retention rate). The 3 individuals dropped out due to hospitalization for exacerbation of hemoptysis, hospitalization for treatment of comorbidities, and catheter infection.

### Acceptability of Program Structure

The responses by the 9 participants who completed the program are shown in Table 4. Furthermore, the following comments were received regarding the program’s structure:

The weekly online real-time program made me feel as though I were back at work again, and it refreshed me and made me feel lively and excited.

The Apple Watch helped me understand my physical condition better through objective numbers and data, and it gave me the opportunity to face my illness properly. Just experiencing symptoms can be vague, and they are easily forgotten, but being able to check the numbers and monitor my condition is really helpful.

I didn’t know anything about mindfulness at first, so I felt hesitant. I think it would be much easier to engage if I could learn why mindfulness is applied to this illness.

I take diuretics every day, so it would be helpful if there were an on-demand option that allows me to participate calmly without worrying about needing to urinate.

**Table 4.** Feedback on the number of sessions, duration per session, and session intervals (n=9).

Variable	Sample, n (%)
Number of programs (8 in total)	
Too many	2 (22)
Appropriate	6 (67)
Too few	1 (11)
Time per session (60 minutes per session)	
Too long	0 (0)
Appropriate	9 (100)
Too short	0 (0)
Program interval (once per week)	
Too long	0 (0)
Appropriate	7 (78)
Too short	2 (22)

### Changes and Awareness After the Program

The qualitative content analysis identified 10 categories (Table 5).

**Table 5.** Qualitative analysis results: categories reflecting changes and awareness after the program.

Category	Frequency, n
Being able to objectively consider one’s thoughts and feelings and not be bothered by them	14
Peace of mind from facing one’s mind and body	13
Reduced pain and handling side effects	12
Being compassionate toward oneself	10
Focusing on one’s body and conducting activities	10
Awareness of the importance of breathing	6
Increased happiness and positive feelings	4
Realization that actions during illness until the present were not erroneous	4
Reduced fatigue	3
Acquiring methods of thinking and coping when feeling anxious or restless	2

#### Being Able to Objectively Consider One’s Thoughts and Feelings and Not Be Bothered by Them

Participants were able to understand that their mental instability was due to their poor physical condition.

I used to struggle to accept it, but now I can think, ‘My physical condition is bad today, so it’s natural [for my feelings to also decline]. It can’t be helped.’ […] And I can tell myself, ‘It’s the illness that makes me feel this way, so it can’t be helped.’

[ID7]

#### Reduced Pain and Handling Side Effects

They experienced how meditation can help relieve pain and heartburn.

I had been having a headache for a long time, but while I was doing the body scan meditation, the pain disappeared… It’s really strange, but the pain seems to move around. I always wonder why.

[ID5]

#### Being Compassionate Toward Oneself

Even when thoughts and feelings came to mind, they were able to observe them objectively and respond more adaptively. Consequently, they felt that this positive change reduced self-blame and harsh self-criticism.

I really felt that the number of times I thought, ‘Oh no, this is terrible,’ had decreased...I really felt that the number of times I thought.

[ID2]

#### Focusing on One’s Body and Conducting Activities

By focusing on bodily sensations and intentionally attending to their internal states during daily activities, participants reported reduced pain.

At night, after eating dinner and taking my medication, my heart used to start pounding faster. But now, after I finish eating, I take a moment and tell myself, ‘Let’s breathe slowly and take a little rest,’ and the breathlessness goes away.

[ID4]

### Secondary Outcomes

**Figure 4.**
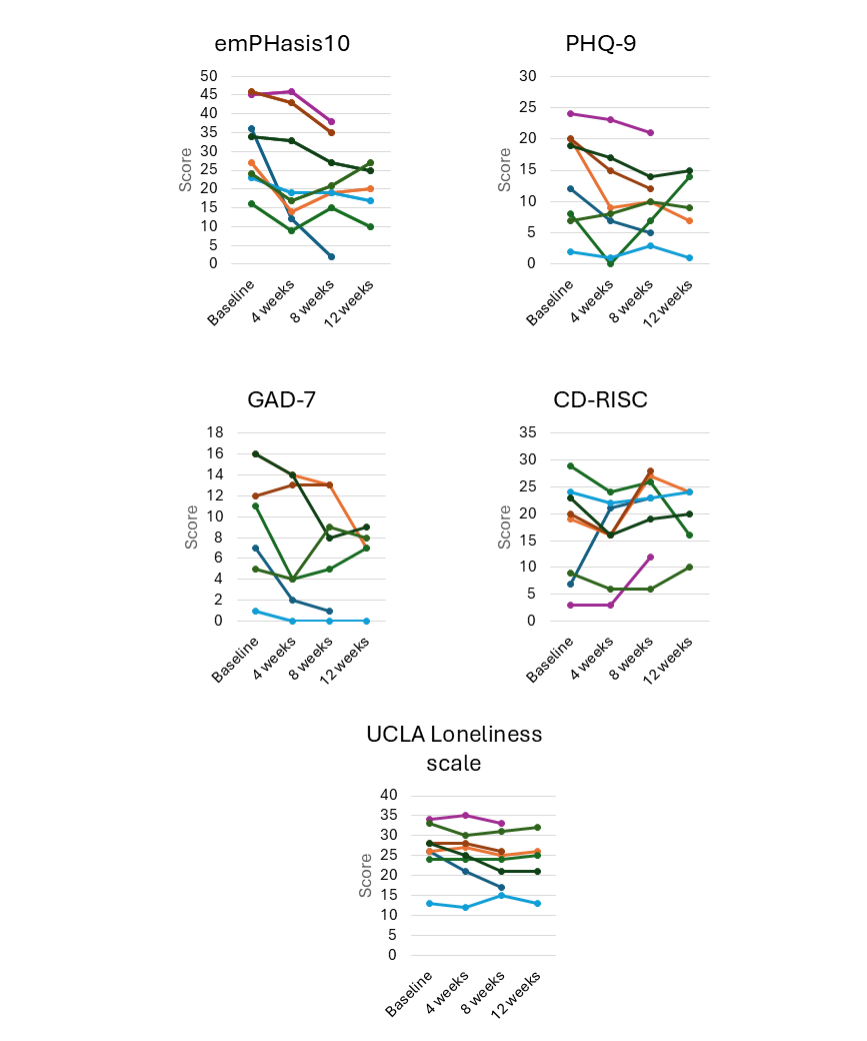
Individual trajectories of patient-reported outcome measures across the four assessment time points (baseline, 4 weeks, 8 weeks, and 12 weeks). The figure displays individual-level changes in 5 psychosocial and clinical measures used in this study: the emPHasis-10 (disease-specific quality of life), PHQ-9 (depressive symptoms), GAD-7 (anxiety symptoms), CD-RISC (resilience), and the UCLA Loneliness Scale (loneliness). Each colored line represents a single participant’s score over time. These descriptive data illustrate variability in symptom patterns and potential trends in psychological well-being during and after the mindfulness-based intervention. CD-RISC: Connor-Davidson Resilience Scale; GAD-7: Generalized Anxiety Disorder 7-item scale; PHQ-9: Patient Health Questionnaire-9.

Although 9 participants completed the program (75% completion rate), the week 8 quantitative analysis—the primary endpoint—included data from 8 participants (67% data retention rate) because one completer did not return the postprogram questionnaire. Figure 4 shows score trends from baseline, week 4, week 8, and week 12. At week 12 (one month after the end of the program), responses were obtained from 5 participants (56% response rate).

The emPHasis-10 (*χ*²_2_=9.74; *P*=.008) and CD-RISC (*χ*²_2_=7.27; *P*=.03) scores showed significant differences at baseline (preprogram), 4 weeks (during the program), and 8 weeks (postprogram; n=8). The significant differences were not maintained at 12 weeks (follow-up). In addition, although no significant difference was found, a trend toward improvement in the PHQ-9 (*χ*²_2_=4.75; *P*=.09) and GAD-7 (*χ*²_2_=5.07; *P*=.08) was observed. Moreover, a few participants showed sudden deterioration in QOL and experienced depressive and anxiety symptoms a month after the program; however, they reported that this coincided with the timing of replacing the subcutaneous Treprostinil injection needle. No significant changes were observed in feelings of loneliness. Individual score trajectories are shown in Figure 4.

## Discussion

### Principal Findings

This study evaluated the feasibility, accessibility, and preliminary psychological changes associated with an online mindfulness-based program designed for patients with PH. The program achieved good retention, with a completion rate of 75% (9/12), and was generally well accepted by participants. Quantitative analyses revealed positive changes in QOL, depression, anxiety, and resilience scores, while qualitative findings indicated that participants experienced greater bodily awareness, emotional regulation, and self-compassion through mindfulness practice. Together, these results suggest that an online mindfulness-based intervention is both feasible and acceptable for patients with PH and may have the potential to improve their psychological well-being and symptom management.

#### Feasibility and Accessibility

With a completion rate of 75% (9/12), the program demonstrated a relatively high level of retention. However, the primary reason for participant dropout was hospitalization due to physical deterioration associated with underlying or comorbid conditions. As PH progresses, patients may develop worsening right heart failure or experience hemoptysis, necessitating hospitalization. Therefore, future implementations of the program should target individuals with stable PH and take into account the potential for physical decline during participation.

The 60-minute session length appeared appropriate; however, participants indicated that the total number of sessions (n=8) was excessive and that the weekly interval was too short. Additionally, some participants expressed a desire for on-demand sessions, citing difficulties caused by frequent urination due to diuretic use. These findings suggest the need to reconsider the program’s frequency and delivery format.

The program consisted of eight 60-minute real-time sessions conducted weekly via an online conferencing system. For PH patients who experience challenges leaving their homes, this online delivery format effectively reduced their physical burden. Nevertheless, the requirement to connect online at a fixed time each week imposed an additional burden that may have negatively affected retention. Conversely, participants who had stopped working due to illness reported a sense of fulfillment and accomplishment from attending regularly scheduled sessions.

As discussed above, offering the entire program on an on-demand basis could further reduce participant burden and improve retention. However, inquiry—a central element of mindfulness—requires real-time interaction between instructors and participants. Inquiry, also referred to as mindful dialog, provides participants with opportunities for self-reflection through guided conversation. To preserve this essential component, at least part of the program should be conducted synchronously rather than fully on-demand. Although traditional MBSR and MBCT programs comprise 8 sessions, recent studies have developed and validated shorter mindfulness-based interventions [37-40]. Based on these findings, we propose a hybrid format that reduces the total number of sessions and delivers approximately half of them on demand. This approach may lessen participant burden while maintaining the feasibility and therapeutic integrity of the intervention. Nevertheless, some patients may require individualized support to manage specific physical symptoms, treatment-related side effects, or emotional distress. Therefore, it may be necessary to consider implementing individual consultations before the program or during the intervention period to provide tailored support for such patients.

Furthermore, this intervention was implemented as an individual program. Expanding individual programs to a larger population poses logistical challenges and limits generalizability. Future studies should therefore examine the effectiveness of group-based formats. In addition, since mindfulness interventions require trained facilitators, the current shortage of qualified personnel represents a barrier to broader dissemination. To promote scalability, two approaches may be considered: (1) developing on-demand mindfulness modules that can be facilitated by health care professionals without formal mindfulness training, or (2) establishing training programs to cultivate health care providers capable of delivering mindfulness interventions.

Although the response rates for pre- and midprogram questionnaires were high, those at program completion and 4 weeks postcompletion were notably low. In this study, paper-based questionnaires were distributed and returned by mail. For PH patients who find it burdensome to leave home, this method likely contributed to the low response rate. Therefore, future studies should employ web-based questionnaires that allow participants to respond online, thereby reducing the response burden and improving data collection rates.

#### Qualitative Findings: Psychological and Physical Experiences During the Program

Qualitative analysis identified themes such as “finding peace of mind through connecting with the body and mind” and “recognizing the importance of breathing.” Interoception refers to perceiving, accessing, and evaluating internal bodily signals [38]. In mindfulness meditation, participants focus nonjudgmentally on present-moment bodily sensations. Rumination arises when attention drifts toward the past or future; mindfulness meditation helps disengage from rumination by redirecting awareness to the present body and activating interoceptive processing [41]. In this program, body-scan and breath-focused meditations enhanced participants’ interoceptive awareness, helping them notice their breathing patterns, bodily reactions during activities, and the thoughts and emotions arising in their minds. Through nonjudgmental observation, participants experienced decentering, which enabled them to view their situations from a distance, thereby reducing rumination and perceived pain.

In addition, lectures and cognitive-behavioral activities addressing emotions and physical symptoms related to treatment side effects provided opportunities to learn coping strategies and methods for engaging with these symptoms. By increasing bodily awareness and practicing mindful engagement during activities, participants reported decreased fatigue and pain. These observations suggest that, in addition to mindfulness practice itself, learning management strategies tailored to PH-related side effects and adaptive coping methods may contribute to reducing treatment-related discomfort and improving activity tolerance.

Breathing was a key element of mindfulness meditation. Before the study, there was concern that focusing on breathing might exacerbate dyspnea in PH patients. Contrary to expectations, meditation focusing on breathing appeared to lessen perceived breathlessness. For individuals experiencing daily dyspnea, increasing awareness of breathing helped them recognize shallow breathing patterns and intentionally breathe more deeply and slowly, which may have contributed to reduced shortness of breath during exertion.

Furthermore, improvements in self-compassion, well-being, and positive emotions were observed after program participation. Many PH patients tend to blame themselves, thinking, “If only I had sought treatment earlier,” or “I became sick because I was weak.” Mindfulness may have helped reduce self-critical thinking by fostering objective awareness of mental states and thought patterns, encouraging nonjudgmental observation, and supporting more positive self-recognition. Overall, these findings suggest that the program may have a favorable influence on mental health and emotional well-being, though further investigation in larger, controlled studies is needed.

### Comparison With Previous Work

Changes observed in QOL, depression, anxiety, and resilience scores suggest that the intervention may have the potential to support improvements in psychological well-being. However, immediate effects on rapidly worsening physical symptoms should not be expected. In this study, standardized self-administered questionnaires were conducted at four time points (baseline, week 4, week 8, and week 12). Because this was a feasibility study with a small sample size and a low response rate at week 12, statistical analyses across all time points were limited. Analyses of the first three time points (baseline, week 4, and week 8) indicated preliminary improvements in QOL, with several participants demonstrating downward trends in depression and anxiety scores. While these findings are not sufficient to determine efficacy, they suggest possible patterns of change that merit further investigation in future controlled trials.

Previous studies of mindfulness-based interventions in cardiovascular populations provide context for interpreting these findings. A randomized controlled trial (The Stress Reduction, Meditation, and Mindfulness Program) targeting patients with chronic heart failure reported reductions in perceived stress and improvements in clinical outcomes [26]. Similarly, a meta-analysis of randomized controlled trials in patients with coronary artery disease demonstrated significant reductions in depression, anxiety, and stress following mindfulness-based interventions [25]. Although the present study focused on patients with PH, the observed trends—increased emotional regulation, decreased rumination, and enhanced self-compassion—are consistent with mechanisms reported in these previous studies.

The mean CD-RISC score in this sample was lower than that reported in patients with cancer (29.3) [42] or multiple sclerosis (26.8) [43], suggesting relatively low baseline resilience in participants. While the small sample size limits interpretation, the observed changes in resilience scores suggest that mindfulness-based programs may hold potential for supporting resilience in PH populations. These preliminary findings align with previous literature indicating that mindfulness practice may contribute to improvements in adaptive coping and emotional well-being across diverse chronic illness populations.

### Limitations

This study has several limitations that should be acknowledged.

First, as a single-arm feasibility study, it did not include a control group. Without a comparison to usual care or another intervention, it is difficult to determine whether the observed improvements in quality of life or psychological measures were attributable to the program itself or to other factors, such as natural adaptation or social interaction with facilitators. To mitigate this, standardized self-report measures were used at multiple time points to examine within-participant changes. Future studies should include a randomized controlled design to allow more robust evaluation of efficacy.

Second, the sample size was small, which limits the statistical power and generalizability of the findings. Because of the limited number of participants, subgroup analyses could not be performed, and some trends may have gone undetected. Nevertheless, the study provided valuable preliminary data on feasibility and participant experiences, which will help inform sample size estimation and stratification criteria for future trials.

Third, the study duration was relatively short, and no long-term follow-up was conducted. Consequently, it remains unclear whether the improvements observed immediately after the intervention can be sustained over time. Future studies should incorporate follow-up assessments to examine the persistence of psychological and physical benefits and to identify factors that influence continued engagement with mindfulness practice.

Fourth, response rates for the postprogram and 4-week follow-up questionnaires were low, possibly because of the paper-based mailing method, which may have introduced response bias. Participants who continued responding may have been those more motivated or satisfied with the program. To improve response rates and minimize bias, future research should use web-based data collection methods to facilitate participation and reduce the burden on patients with PH.

### Future Directions

Based on these findings, several future directions for research and program development can be proposed. First, future studies should conduct randomized controlled trials with larger and more diverse samples to verify the program’s efficacy and assess its long-term impact. Longitudinal follow-up is necessary to evaluate whether the psychological and physical benefits observed in this feasibility study can be sustained over time. Multisite or decentralized clinical trial designs may also facilitate participation among patients with PH who face difficulties traveling to research facilities.

Second, refinement of the program content is warranted. Adjustments to session frequency and duration, as well as partial incorporation of on-demand components, may help reduce participant burden and improve accessibility. Developing a group-based version of the program could further enhance social connectedness and scalability.

Third, to promote broader dissemination and generalization of mindfulness-based interventions, it will be important to develop and implement educational programs that train cardiovascular health care professionals to practice and facilitate mindfulness. Increasing the number of qualified practitioners is expected to contribute to the sustainable delivery and expansion of such programs in clinical and community settings. In addition, future efforts should focus on expanding the availability of mobile apps across different digital platforms and, ultimately, adapting and implementing the program in international settings. Such developments may facilitate broader accessibility and cross-cultural validation of mindfulness-based interventions for patients with chronic cardiopulmonary diseases.

### Conclusions

This feasibility study suggests that an online mindfulness-based self-management program may help patients with pulmonary hypertension engage more effectively with symptoms, treatment-related side effects, and emotional distress. Participants reported preliminary improvements in perceived pain and aspects of quality of life, indicating potential psychological benefits. Further refinement of the program and evaluation in larger randomized controlled trials are needed to determine its efficacy and long-term impact.

## Abbreviations

CD-RISC: Connor-Davidson Resilience Scale
GAD-7: Generalized Anxiety Disorder 7-item scale
MBCT: mindfulness-based cognitive therapy
MBSR: mindfulness-based stress reduction
PAH: pulmonary arterial hypertension
PH: pulmonary hypertension
PHQ-9: Patient Health Questionnaire-9
QOL: quality of life
